# Identification of a ceRNA Network in Lung Adenocarcinoma Based on Integration Analysis of Tumor-Associated Macrophage Signature Genes

**DOI:** 10.3389/fcell.2021.629941

**Published:** 2021-03-02

**Authors:** Lei Zhang, Kai Zhang, Shasha Liu, Ruizhe Zhang, Yang Yang, Qi Wang, Song Zhao, Li Yang, Yi Zhang, Jiaxiang Wang

**Affiliations:** ^1^Department of Surgery, The First Affiliated Hospital of Zhengzhou University, Zhengzhou, China; ^2^Biotherapy Center, The First Affiliated Hospital of Zhengzhou University, Zhengzhou, China; ^3^Reproductive Medicine Center, The First Affiliated Hospital of Zhengzhou University, Zhengzhou, China

**Keywords:** tumor-associated macrophages, lung adenocarcinoma, LASSO cox regression, WGCNA, competing endogenous RNA

## Abstract

As research into tumor-immune interactions progresses, immunotherapy is becoming the most promising treatment against cancers. The tumor microenvironment (TME) plays the key role influencing the efficacy of anti-tumor immunotherapy, in which tumor-associated macrophages (TAMs) are the most important component. Although evidences have emerged revealing that competing endogenous RNAs (ceRNAs) were involved in infiltration, differentiation and function of immune cells by regulating interactions among different varieties of RNAs, limited comprehensive investigation focused on the regulatory mechanism between ceRNA networks and TAMs. In this study, we aimed to utilize bioinformatic approaches to explore how TAMs potentially influence the prognosis and immunotherapy of lung adenocarcinoma (LUAD) patients. Firstly, according to TAM signature genes, we constructed a TAM prognostic risk model by the least absolute shrinkage and selection operator (LASSO) cox regression in LUAD patients. Then, differential gene expression was analyzed between high- and low-risk patients. Weighted gene correlation network analysis (WGCNA) was utilized to identify relevant gene modules correlated with clinical characteristics and prognostic risk score. Moreover, ceRNA networks were built up based on predicting regulatory pairs in differentially expressed genes. Ultimately, by synthesizing information of protein-protein interactions (PPI) analysis and survival analysis, we have successfully identified a core regulatory axis: LINC00324/miR-9-5p (miR-33b-5p)/GAB3 (IKZF1) which may play a pivotal role in regulating TAM risk and prognosis in LUAD patients. The present study contributes to a better understanding of TAMs associated immunosuppression in the TME and provides novel targets and regulatory pathway for anti-tumor immunotherapy.

## Introduction

As reported in World Cancer Report 2020, lung cancer continues to be the most common cancer type and the leading cause of cancer death worldwide, accounting for about 18% of all cancer deaths. Non-small-cell lung cancer represents 80–85% of lung cancers, and can be subdivided into adenocarcinoma, squamous-cell carcinoma, and large-cell carcinoma, etc (Reck and Rabe, 2017; Siegel et al., 2020). Among them, lung adenocarcinoma (LUAD) is more aggressive and possesses more morphological heterogeneity than other types of lung cancer (Zhang et al., 2018).

In spite of advances in chemotherapy, radiotherapy, and targeted therapy in the last decade, prognosis of patients with advanced lung cancer is still very poor, with a 5-year survival rate of 10–20% (Allemani et al., 2015). As research progresses, immunotherapy is becoming the most promising treatment against cancers, and has gradually revolutionized cancer treatment (Herbst et al., 2018). Although several tumor types including LUAD reveal effective response to immunotherapy especially immune checkpoint blockade, it remains a large portion of patients failed to benefit from the treatment (Chen and Mellman, 2017; Hirsch et al., 2017).

Recent studies have demonstrated that the tumor microenvironment (TME) plays the key role influencing the efficacy of anti-tumor immunotherapy, in which tumor-associated macrophages (TAMs) are the most important component (Bohn et al., 2018; Xia et al., 2020). TAMs are abundant in multiple cancers compared to adjacent tissues, supporting oncogenesis, vascularization in spite of immunosurveillance (Yang and Zhang, 2017). This raises the intriguing possibility that targeting TAMs may be an effective therapeutic strategy for intractable LUAD (Cassetta and Pollard, 2018). Actually, considering the extremely complicated microenvironment, TAMs appear to be highly heterogeneous in solid tumors. Meanwhile, TAM-associated molecular markers appear to show controversial prognostic value in pan-cancer patients (Pollard, 2004; Lu-Emerson et al., 2013; Ojalvo et al., 2018; Wang et al., 2018; Cao et al., 2019; Li et al., 2019; Liu et al., 2019; Dai et al., 2020). Collective evidence had previously demonstrated that TAMs were characterized mostly by M2-like markers and were correlated with poor prognosis in numerous malignancies, including lung cancer. Therefore, we assumed that identification of M2-like TAMs risk was more meaningful because they are primarily responsible for the prognosis of patients.

Accumulating evidences have emerged revealing competing endogenous RNAs (ceRNAs) play an essential role in regulating interactions among different varieties of RNAs and were involved in progression and immune infiltration in multiple kinds of tumors (Zhang K. et al., 2020). However, there is limited comprehensive investigation focusing on the regulatory mechanism between ceRNA networks and TAM associated signature genes, and the deep prognostic value is not yet fully explored. In this study, we aimed to utilize bioinformatic approaches analyzing public datasets to explore how TAMs potentially influence the prognosis of LUAD patients and tried to provide novel targets and directions for anti-tumor immunotherapy, especially for targeting the TAMs.

## Materials and Methods

### Study Design

**FIGURE 1:**
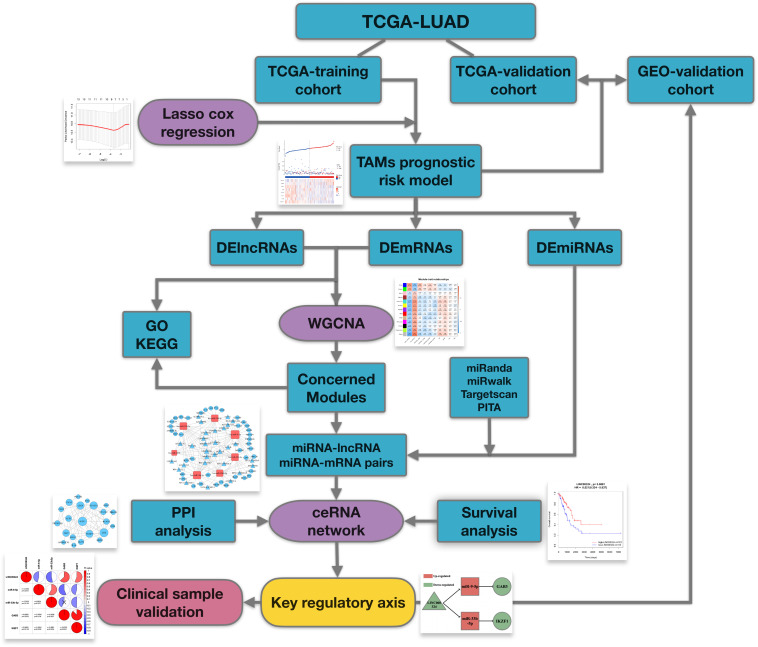
Flowchart of this work.

The workflow of the manuscript is shown in Figure 1. Public datasets TCGA-LUAD and an external GEO validation cohort, containing sequencing data of LUAD tumor tissues, were analyzed in this study. As for TCGA-training cohort, utilizing TAMs associated genes, we firstly constructed a TAMs prognostic risk model by LASSO cox regression. Samples were divided into high- and low-risk groups according to their calculated risk scores. Then differentially expressed genes between two groups were identified for following comprehensive analysis. WGCNA was utilized to identify relevant gene modules correlated with clinical characters and prognostic risk score and ceRNA networks were then built up in concerned WGCNA modules. At last, by synthesizing information of PPI analysis and survival analysis, we tried to identify a core regulatory axis in ceRNA networks which may play a key role in TAMs associated immunosuppression and prognostic value in LUAD patients.

### Public Data Used in This Study

The TCGA-LUAD data, containing expression value of lncRNAs, miRNAs, and mRNA as well as clinical information were retrieved from the TCGA data portal^1^. Besides, another external validation set GSE72094, defined as GEO-validation cohort was obtained from the GEO website^2^. After removing patients who have ever been affected by other malignant tumors or with incomplete lncRNAs, miRNAs, and mRNAs data, 804 LUAD patients were screened for inclusion in this study. Expression value of genes was then standardized for subsequent analysis.

### Construction of TAMs Related Prognostic Risk Model

According to comprehensively reported TAMs related gene signature: CD68, CD11b, CD163, CD206, IL10, CD39, MMP14, CXCL8, CCL17, CD274, TGFB1, ARG1, and IDO1, a TAMs related prognostic risk model was built up by the least absolute shrinkage and selection operator (LASSO) cox regression (Duan et al., 2016). Package “glmnet” in R software (version 3.3.2) was utilized to achieve this result. Optimized lambda was determined when the cross-validation error reach to the smallest and non-zero coefficients were selected. Subsequently, a risk score was built according to the expression value and module coefficient of each gene (Lossos et al., 2004; Chen et al., 2007; Hu et al., 2019):

$$\mathrm{Risk}\,\mathrm{score}=\sum_{i=1}^{k}\beta \,i\,S\,i$$

where k, βi, and Si represent the number of signature genes, the coefficient index, and the gene expression level, respectively. Subsequently, a risk score was built according to the expression value and module coefficient of each gene and therefore patients were assigned into high-risk group and low-risk group based on the risk score. At last, Kaplan-Meier survival analysis were performed to validate prognostic value of the risk model.

### Survival Analysis

The univariate Cox regression model was applied to analyze the relationship between the overall survival (OS) of LUAD patients and gene expression. *p*-value < 0.05 was considered to be significant. Then, the selected genes were visualized with the Kaplan-Meier survival curve. Time-dependent ROC analyses were conducted by “timeROC” R package to estimate the accuracy of the predicted survival probability.

### Identification of Differentially Expressed Genes

Random variance model (RVM) *t*-test was applied to filter the differentially expressed genes among groups. After the significant analysis and FDR analysis, we selected the differentially expressed genes according to the criterion: FDR < 0.05 and absolute FC > 1.2. Then, the relative abundances of differentially expressed lncRNAs (DElncRNAs), differentially expressed miRNAs (DEmiRNAs) and differentially expressed mRNAs (DEmRNAs) were illustrated in heatmaps by R package “gplots.”

### Weighted Gene Correlation Network Analysis

In order to analyze potential interconnections between DEGs, Weighted gene correlation network analysis (WGCNA) analysis was employed to identify modules containing genes with similar expression patterns via the package “WGCNA” in R software (Langfelder and Horvath, 2008). Firstly, cluster analysis was performed to remove outliers based on differential gene expression of samples. Next, to balance the relationship between scale independence and mean connectivity, a suitable soft-threshold power β was determined. Then Topological Overlap Matrix (TOM) was constructed based on β value, deriving the intergenic divergence coefficients. Cluster Dendrogram and Eigengene Adjacency Heatmap were drew to show gene clustering and module relationship. To determine the most important module for further analysis, eigengene for each module was calculated. Then eigengenes were employed to compute module-trait associations with risk score, TNM stage, age, gender, race, and RFS, etc. Finally, concerned modules were identified by considering genes numbers and association between modules and clinical features.

### Gene Ontology and KEGG Pathway Enrichment Analysis

Gene ontology (GO) analysis was applied to analyze the main function of genes according to the Gene Ontology database^3^, which can organize genes into hierarchical categories and uncover the gene regulatory network on the basis of biological process and molecular function. The Kyoto Encyclopedia of Genes and Genomes (KEGG)^4^ (Kanehisa et al., 2010) was used to analyze the potential regulatory pathways of DEG and genes involved in concerned WGCNA modules. The functional annotations were performed by “clusterProfiler” package in R software.

### Construction of ceRNA Networks

To predict the possible target mRNAs and lncRNAs of DEmiRNAs, several target gene prediction websites were utilized. Firstly, by searching miRanda^5^, Targetscan^6^ (Lewis et al., 2005), and miRWalk^7^ databases, we got intersection of predicted miRNA-mRNA pairs with possible binding relation. Similarly, miRNA-lncRNA pairs were predicted by employing PITA^8^ and miRanda databases. Then, miRNA-mRNA/miRNA-lncRNA pairs with negatively correlation were finally determined in concerned WGCNA modules separately. Ultimately, the ceRNA networks were constructed by integrating the miRNA–lncRNA-mRNA interactions by Cytoscape 3.4.0 software (Lotia et al., 2013).

### Protein-Protein Interactions (PPI) Networks Analysis

The protein-protein interactions (PPI) between mRNAs in ceRNA networks were constructed by Search Tool for the Retrieval of Interacting Genes (STRING) database^9^ (Szklarczyk et al., 2011). Interactions with confidence score ≥0.4 were demonstrated in the networks.

### Quantification of Genes by Quantitative Real-Time PCR

A total of 20 surgical resection of tumor tissues from LUAD patients were obtained from the Thoracic Surgery department of the First Affiliated Hospital of Zhengzhou University. Informed consents were signed by patients before surgeries, and this research was approved by the Institutional Ethical Committee of the First Affiliated Hospital of Zhengzhou University. Total RNA was extracted from tumor tissues with TRIzol reagent (Invitrogen, United States). The purity and concentration of RNA were quantified by NanoDrop 2000 spectrophotometer (Thermo Fisher Scientific, United States). For mRNA and lncRNA detection, 1 mg total RNA was used to synthesize the first strand cDNA using PrimeScript RT reagent Kit With gDNA Eraser (TaKaRa, Japan). For miRNA detection, cDNA was synthesized with miRNA First Strand cDNA Synthesis (Tailing Reaction) (Sangon, China). qRT-PCR were carried out using SYBR Premix Ex Taq II (TaKaRa, Japan) in CXF96 System (BioRad, United States). GAPDH and U6 were used as endogenous control. The primers used in this study were listed in Supplementary Table 5.

## Results

### Information of Samples and TAMs Biomarkers Enrolled in This Study

The flowchart in Figure 1 demonstrates the overall design and process about this study. To begin with, we have screened suitable samples for analysis by removing patients who have ever been affected by other malignant tumors and only samples with expression data of lncRNA, miRNA and mRNA could be enrolled. Firstly, we randomly assigned 406 samples from TCGA-LUAD into two groups, 203 in TCGA-training cohort, and 203 in TCGA-validation cohort. Besides, another external validation set GSE72094, containing 398 LUAD samples, was defined as GEO-validation cohort. In total, 804 LUAD patients were screened for inclusion in this study. As for TAM biomarkers, 13 candidate genes which have been comprehensively reported associated with phenotype or function of TAMs were enrolled in analysis, including CD68, CD11b, CD163, CD206, IL10, CD39, MMP14, CXCL8, CCL17, CD274, TGFB1, ARG1, and IDO1, and the details about these TAM-related genes were showed in Supplementary Table 1.

### Construction of a Prognostic Risk Model Based on TAM Signature Genes

**FIGURE 2:**
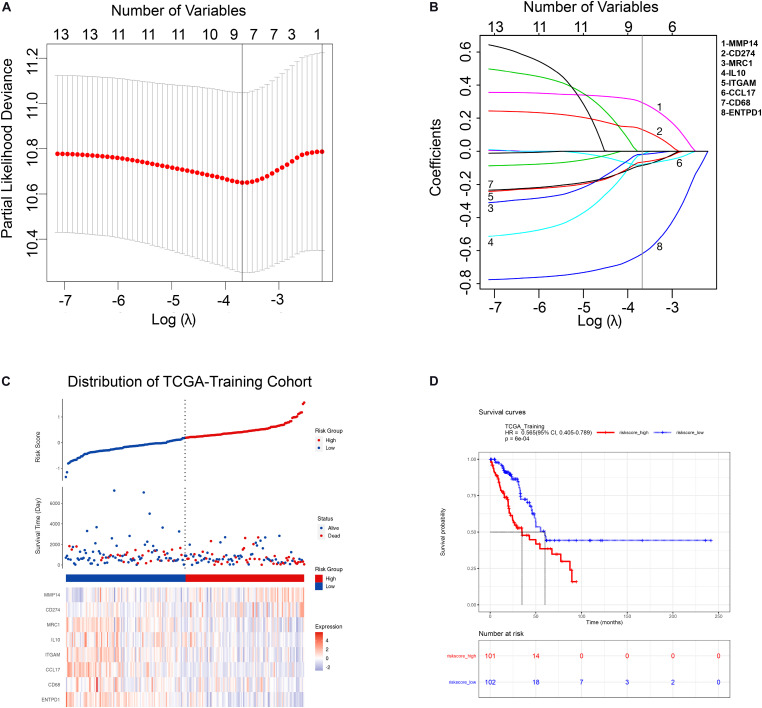
Construction of TAM risk model in TCGA-training cohort. **(A)** Optimized lambda determined in the LASSO regression model. A vertical line is drawn at the value chosen by 10-fold cross-validation. **(B)** The LASSO coefficient spectrum of the 13 TAM-related genes. **(C)** Expression heatmap of identified 8 genes and corresponding risk score as well as survival status of patients in TCGA-training cohort. **(D)** KM survival curve for high- and low-risk group patients in TCGA-training cohort.

In order to comprehensively assess the relationship between TAM-related genes and the prognosis of LUAD patients, a LASSO cox regression model was used in TCGA-Training cohort to calculate the most valuable prognostic genes. The optimized lambda determined in Figure 2A was utilized to select features with non-zero coefficients, and the LASSO coefficient profiles of TAM-associated genes are shown in Figure 2B. Based on the LASSO regression model and the prognosis of patients, 8 potential predictors were screened in the TCGA-Training cohort: CD68, ITGAM, MRC1, IL10, CD274, ENTPD1, CCL17, and MMP14. Subsequently, a risk score was built according to the expression value and module coefficient of each gene: Risk Score = (−0.085962 × CD68 expression) + (−0.065982 × ITGAM expression) + (−0.018361 × MRC1 expression) + (−0.019623 × IL10 expression) + (0.132375 × CD274 expression) + (−0.620876 × ENTPD1 expression) + (−0.080381 × CCL17 expression) + (0.295009 × MMP14 expression). Then, risk score for each patient in TCGA-Training cohort was calculated using this formula, and therefore patients were assigned into high-risk group and low-risk group according to the median of the risk score (Figure 2C). Univariate and multivariate analyses suggested that TAMs risk score in this study was an independent prognostic factor of LUAD patients (Supplementary Table 6). Kaplan-Meier survival analyses showed that the prognosis of patients in the high-risk group was significantly poor than that in the low-risk group (Figure 2D), indicating that the risk model we constructed had a predictive role in the prognosis of LUAD patients.

### TAM Risk Model Displays a Consistent Predictive Capacity in Validation Cohort

**FIGURE 3:**
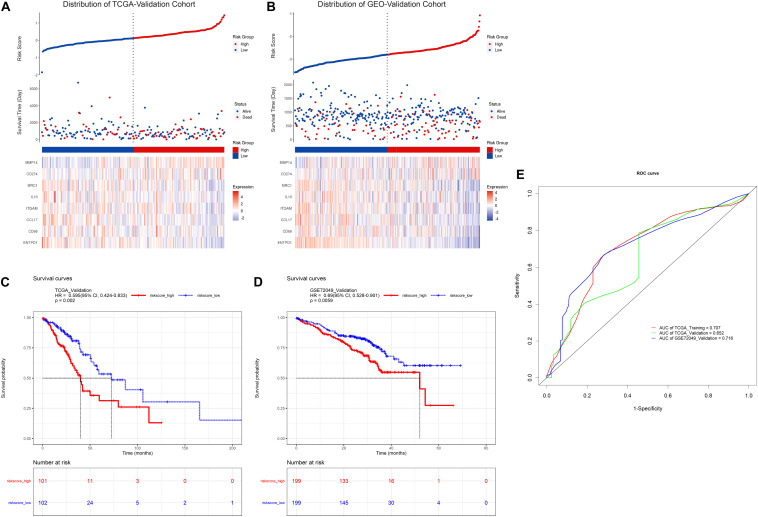
Validation of TAM risk model in validation cohorts. Expression heatmap as well as survival status of patients in TCGA- or GEO-validation cohort are showed in **(A,B)**. KM survival curve for high- and low-risk group patients in TCGA- or GEO-validation cohort are showed in **(C,D)**. **(E)** Time-dependent ROC curve of risk score in three cohorts.

To further evaluate the prognostic value of the risk model constructed above, similarly, the risk score formula was employed to calculate risk score for patients in TCGA-validation and GEO-validation cohort. As a result, the distribution of patients with risk score as well as prognostic status was presented in Figures 3A,B, respectively. In addition, Kaplan-Meier survival analyses in Figures 3C,D demonstrated that patients with higher risk score in these two validation sets tend to possess a worse prognosis which was consistent with the results in TCGA-training set. Moreover, time-dependent receiver operating characteristic curve (ROC) was plotted for predictive capacity of the risk model in Figure 3E. From the figure we can see that the area under curve (AUC) for TCGA-training, TCGA-validation and GEO-validation was 70.7, 65.2, and 71.6% respectively. The results suggest that the risk model we constructed based on TAM signature genes reveals a good prognostic value.

### Analysis of Differentially Expressed Genes Based on Risk Score

So far, we have successfully constructed a prognostic risk model in LUAD patients based on the expression of TAM-related genes. Afterward, in order to get a deeper insight into the specific molecular mechanisms that induce differences in survival prognosis between high- and low-risk samples, comprehensive analysis about differentially expressed genes between these two groups will be conducted in the following sections.

**FIGURE 4:**
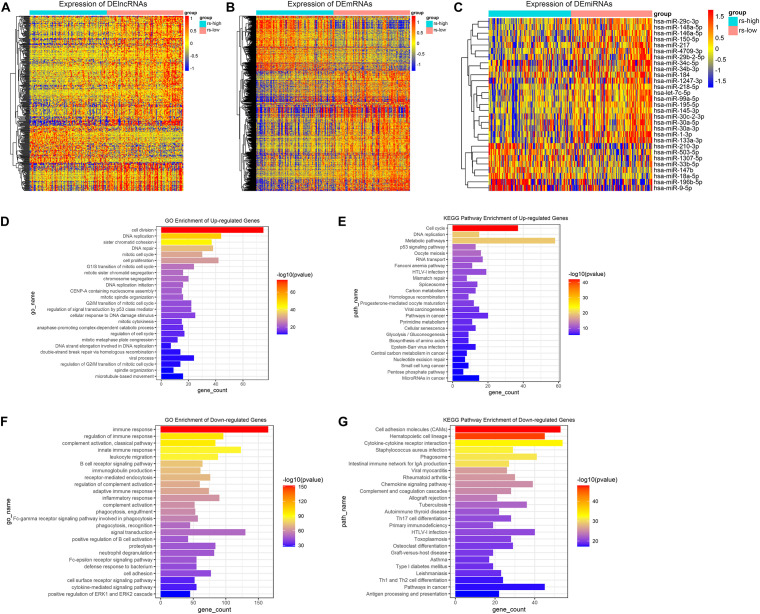
Differential gene expression analysis and function annotation. Heatmaps demonstrate expression of DElncRNAs **(A)**, DEmRNAs **(B),** and DEmiRNAs **(C)**. The top 25 enriched GO biological processes **(D)** and KEGG pathways **(E)** of the significantly upregulated genes. The top 25 enriched GO biological processes **(F)** and KEGG pathways **(G)** of the significantly downregulated genes.

Firstly, differential gene expression analysis was performed between high- and low-risk groups in TCGA-training cohort. Genes with FDR < 0.05 and absolute FC > 1.2 were considered to be significantly changed in expression. Based on this criterion, as a result, we have identified 381 DElncRNAs, 29 DEmiRNAs, and 1976 DEmRNAs between the two groups. Compared to the low-risk group, 81 lncRNAs, 8 miRNAs and 620 mRNAs were upregulated, whereas 300 lncRNAs, 21 miRNAs and 1356 mRNAs were downregulated in the high-risk group. The relative abundances of these genes were illustrated in heatmaps by clustering analysis (Figures 4A–C). As mRNA encoded proteins usually perform major biological functions, biological process and pathway enrichment analysis of DEmRNAs were conducted according to Gene Ontology and KEGG databases. As depicted in Figure 4D, enrichment results showed that upregulated DEmRNAs in high-risk group were primarily involved in GO biological processes (GO-BP), such as “cell division,” “cell proliferation,” “DNA replication” and “DNA repair.” Meanwhile, KEGG pathways analysis also showed that upregulated DEmRNAs were primarily involved in “cell cycle,” “DNA replication” and “metabolic pathways” which were related to tumor development and progression (Figure 4E). On the contrary, regarding to downregulated DEmRNAs in the high-risk group, it is noteworthy that downregulated genes were mainly enriched in “immune response,” “phagocytosis,” and “cytokine-cytokine receptor interaction” (Figures 4F,G). These results collectively demonstrate that DEmRNAs between high- and low-risk groups play a key role in tumor development and immunosuppression which is exactly consistent with the immunosuppression of TAMs in the TME.

### Weighted Gene Correlation Network Analysis Reveals Potential Interconnections Between Differentially Expressed Genes

**FIGURE 5:**
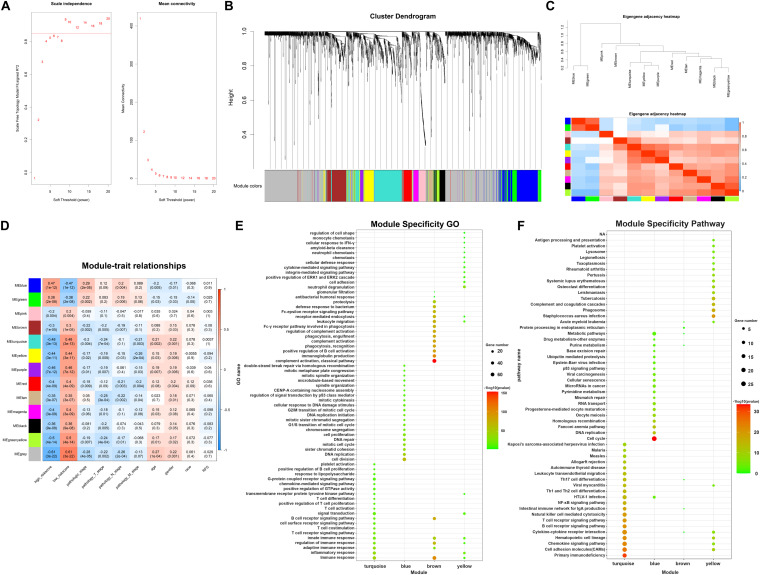
WGCNA analysis identifying concerned modules. **(A)** Determination of soft-threshold power in the lncRNAs/mRNAs WGCNA. Left: Analysis of scale independence for various soft-thresholding powers. Right: Analysis of the mean connectivity for various soft-thresholding powers. **(B)** Cluster dendrogram was generated by hierarchical clustering based on dissimilarity measure of genes. The branches correspond to modules of highly interconnected groups of genes. **(C)** Heatmap plot of the adjacencies in the hub gene network, red represents positive correlation with high adjacency, while blue color represents negative correlation with low adjacency. **(D)** Matrix of module-trait relationships and *p*-values for selected traits. Each row corresponds to a module eigengene, and each column corresponds to a clinical character, each module contains a corresponding correlation value R and *p*-value. **(E)** Module specificity GO enrichment analysis of 4 concerned modules. **(F)** Module specificity KEGG pathway enrichment analysis of 4 concerned modules.

In the following part, in order to analyze the potential interconnections between DEGs, WGCNA analysis was employed to identify modules containing genes with similar expression patterns. Firstly, cluster analysis was performed to remove outliers based on differential gene expression of samples (Supplementary Figure 1). Next, the expression profiles of 381 DElncRNAs and 1976 DEmRNAs were obtained for constructing the co-expression network via the package “WGCNA” in R software. To balance the relationship between scale independence and mean connectivity, a suitable soft-threshold power β should be determined for following construction of WGCNA network. We analyzed the network topology with soft-threshold power from 1 to 20 and finally confirmed β values of 9 in lncRNAs/mRNAs co-expression network analysis (Figure 5A). Then we constructed Topological Overlap Matrix (TOM) based on β value, deriving the intergenic divergence coefficients. From the Cluster Dendrogram in Figure 5B, we could find that genes with similar expression patterns were grouped into modules with specific color. Correlation between modules were showed in Eigengene Adjacency Heatmap (Figure 5C), and there were no modules with too much similarity needed to be merged. Ultimately, a total of 12 modules were generated in the lncRNAs/mRNAs co-expression network, clustering in size from 36 to 341 genes (Supplementary Table 2). The gray module represented a gene set containing genes not suitable for assigning to any other modules. Having assigned DEGs into different color modules, we then want to explore the correlation between modules and clinical characteristics and phenotypes of samples. As shown in Figure 5D, several modules correlated with clinical characteristics, such as age, gender, race, and TNM stage as well as TAM-related risk score. Modules that are positively related to TAM risk score, such as blue module and green modules, tend to be positively related to tumor pathology stage as well. On the contrary, modules negatively associated with TAM risk score, such as turquoise and yellow modules, are more likely related to an earlier pathology stage. These results are accordant with previously reported influence of TAMs on tumor progression. In order to choose suitable modules for following construction of ceRNA networks, we preferred to choose WGCNA modules which contained more lncRNAs/mRNAs co-expression pairs and correlated with clinical features. According to this criterion, the four interested modules that contained the highest number of genes were turquoise, blue, brown, and yellow module (containing 341, 264, 161, and 126 genes respectively). Meanwhile, these concerned modules revealed correlations with clinical stage and TAM risk score. Details about these four modules were enclosed in Supplementary Table 3. Moreover, module specificity GO enrichment analysis and module specificity KEGG pathway enrichment analysis were performed in Figures 5E,F. Of note, turquoise module had a significant correlation with immunosuppression while blue module tended to be associated with tumor development. Overall, employing weighted gene correlation network analysis, we have identified four concerned modules containing co-expressed genes which may play an important role in immunosuppression and tumor development.

### Construction of ceRNA Networks for Concerned WGCNA Modules

**FIGURE 6:**
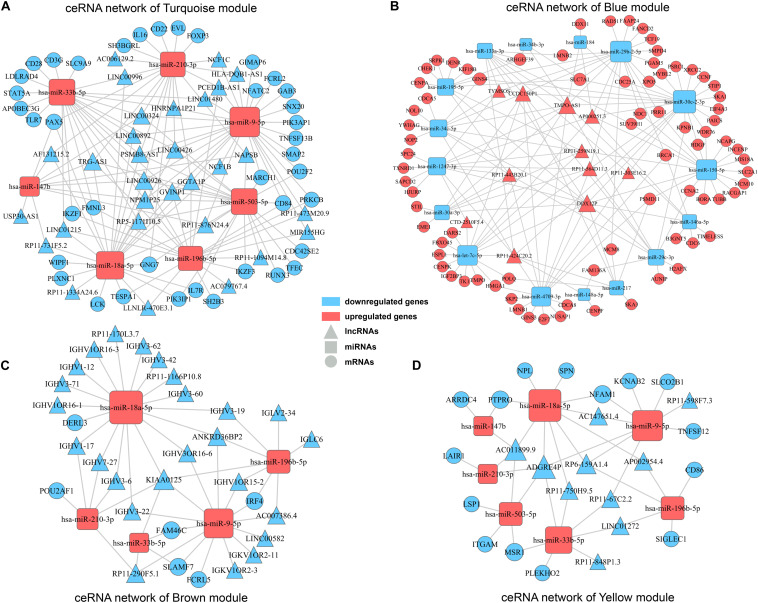
ceRNA networks for concerned WGCNA modules. ceRNA networks were built up for turquoise module **(A)**, blue module **(B)**, brown module **(C),** and yellow module **(D),** respectively. Triangle represents lncRNAs, square represents miRNAs and round represents mRNAs. Blue color indicates downregulated genes, while red color indicates upregulated genes.

Accumulating evidences have emerged revealing that ceRNA theory plays an essential role in explaining interactions among different varieties of RNAs. Briefly, lncRNAs can share miRNA response elements to affect miRNA affinity with target mRNAs, thus regulating gene expression at the transcriptional level. Considering the concerned modules in WGCNA mainly contained lncRNAs and mRNAs with positively correlation, according to the ceRNA theory, there should present miRNAs negatively correlated with lncRNAs and mRNAs, and then collectively forms a ceRNA network. Firstly, by searching DEmiRNAs in miRanda, Targetscan and miRWalk databases, we got predicted miRNA-mRNA pairs with possible binding relation. Similarly, miRNA-lncRNA pairs were predicted by employing miRanda and PITA databases. Then, according to the predicted miRNA-mRNA/miRNA-lncRNA pairs and expression pattern of genes in clinical samples, miRNA-mRNA/miRNA-lncRNA pairs with negatively correlation were finally determined in concerned WGCNA modules separately. Ultimately, ceRNA networks for concerned modules were constructed by integrating the miRNA-lncRNA-mRNA interactions by Cytoscape software (Figure 6). The ceRNA network for turquoise module and blue module involved the most abundant regulatory relationships. There were 30/11 lncRNAs, 7/16 miRNAs, and 39/77 mRNAs in ceRNA network for turquoise module and blue module, respectively. As mentioned before, turquoise module contained genes appear to be associated with immune response and all of these lncRNAs and mRNAs were downregulated in high TAM risk group. Meanwhile, blue module contained genes, upregulated in the high-risk group, were related to tumor development. Therefore, ceRNA networks in these two concerned modules may play a key role in TAMs risk and LUAD prognosis which deserves further analysis.

### Identifying Core Regulatory Axis in ceRNA Network by Synthesizing Information of PPI Analysis and Survival Analysis

**FIGURE 7:**
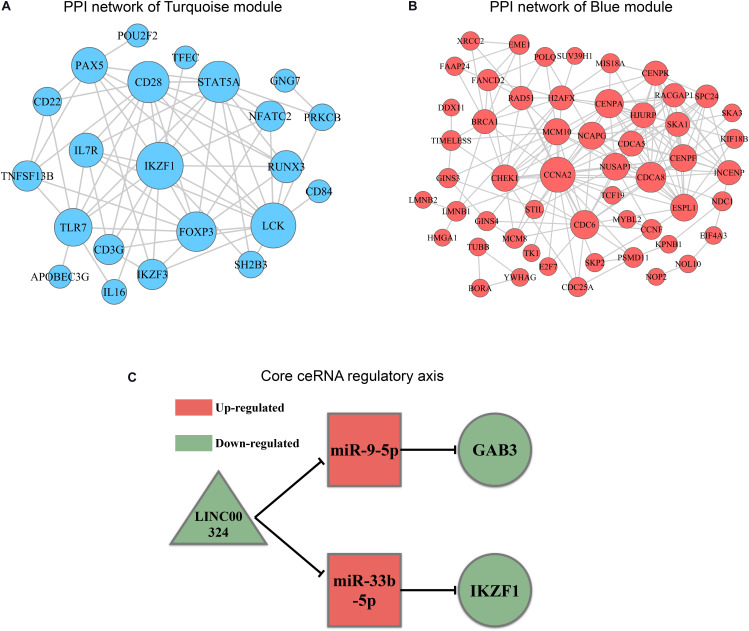
PPI analysis and core regulatory axis in ceRNA network. **(A)** mRNAs involved in ceRNA for turquoise module were utilized to construct a PPI network. **(B)** mRNAs involved in ceRNA for blue module were utilized to construct a PPI network. **(C)** Core regulatory axis identified in this study. Triangle represents lncRNAs, square represents miRNAs and round represents mRNAs.

In order to explore intrinsic relationships between proteins encoded by mRNAs in ceRNA networks, PPI networks were constructed by employing STRING database. We identified hub genes among these DEmRNAs involved in ceRNA networks (Figures 7A,B). Hub genes with top node degree in turquoise module were IKZF1, LCK, CD28, STAT5A, FOXP3, TLR7, PAX5, IL7R, RUNX3, and IKZF3. As for blue module, CCNA2, CHEK1, CDC6, CDCA8, NCAPG, CENPF, NUSAP1, CENPA, MCM10, and HJURP may act as hub genes in the ceRNA network. In the meanwhile, Kaplan-Meier survival analysis was performed for all of the genes involved in ceRNA networks to identify key prognostic genes. As a result, we finally identified 127 genes with prognostic value from the ceRNA networks, including 34 lncRNAs, 7 miRNAs, and 86 mRNAs. Survival analysis results as well as ceRNA regulatory relationships in Turquoise and Blue modules could be find in Supplementary Table 4. According to the intersection of survival analysis results and ceRNA regulatory relationship, we tried to identify ceRNA regulatory axis containing genes with the best prognostic value. Ultimately, by synthesizing information of PPI analysis and survival analysis, we have successfully identified a core regulatory axis: LINC00324/miR-9-5p (miR-33b-5p)/GAB3 (IKZF1) (Figure 7C) which may play a pivotal role in regulating TAM risk and the prognosis in LUAD patients.

### External Validation of the Core Regulatory Axis in GEO Dataset and Clinical Samples

**FIGURE 8:**
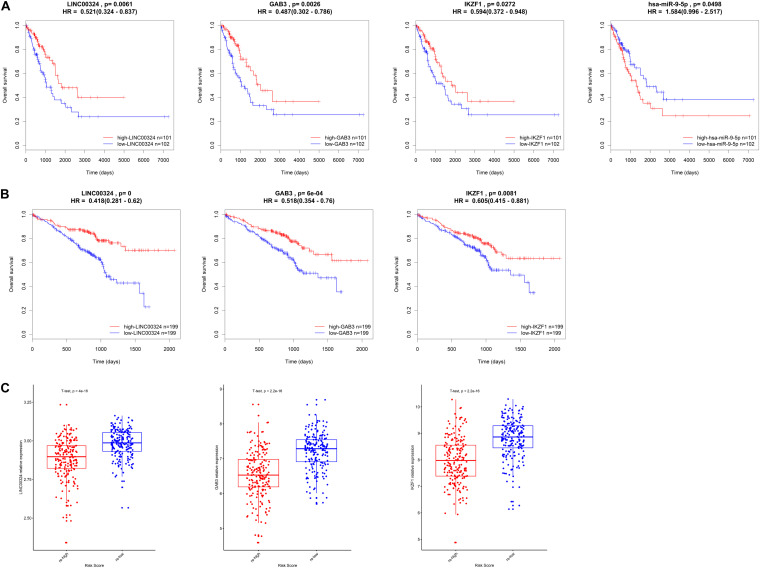
Validation of the core regulatory axis. Survival analysis of LINC00324, GAB3, IKZF1 and miR-9-5p in TCGA database **(A)** or GEO database **(B)**. **(C)** Expression validation of LINC00324, GAB3, and IKZF1 between high- and low-risk groups in GEO cohort.

In order to validate the prognostic value and expression significance of key genes involved in the regulatory axis we identified above, survival analysis and expression analysis were also performed in an external GEO database (Samples were divided into two groups according to the median of expression of genes). As shown in Figures 8A,B, all of LINC00324 (HR = 0.418, *p* < 0.01), GAB3 (HR = 0.518, *p* < 0.01), and IKZF1 (HR = 0.605, *p* < 0.01) could predict a good prognosis in GEO LUAD patients, which were coincident with the result in TCGA database (HR = 0.521, *p* < 0.05 for LINC00324; HR = 0.487, *p* < 0.05 for GAB3; HR = 0.594, *p* < 0.05 for IKZF1). Due to the lack of miRNAs data in GEO database, miR-9-5p only showed prognostic significance in TCGA dataset (HR = 1.584, *p* < 0.05). Then expression difference analysis was carried out in GEO dataset. According to TAM risk model employed before, patients were divided into high- and low-risk group, as expected, all of these three genes were more highly expressed in low-risk group (*p* < 0.01), which indicating their potential role in anti-tumor immunity as mentioned before (Figure 8C).

**FIGURE 9:**
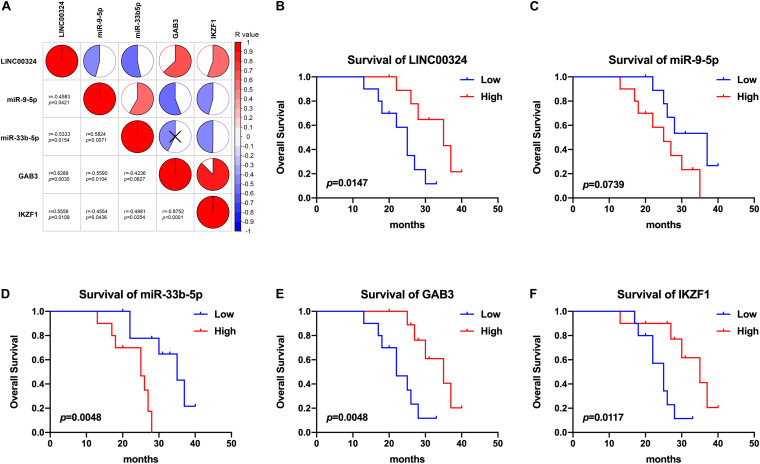
Correlation and survival analysis of the core regulatory axis in clinical samples. **(A)** Correlations between LINC00324, miR-9-5p, miR-33b-5p, GAB3, and IKZF1. Results were analyzed according to gene relative expression achieved by qRT-PCR and correlations were tested using Pearson correlation coefficient. Kaplan-Meier analyses of overall survival in LUAD patients with low (*n* = 10) and high (*n* = 10) LINC00324 **(B)**, miR-9-5p **(C)**, miR-33b-5p **(D)**, GAB3 **(E),** and IKZF1 **(F)** expression.

In addition, small size validation with clinical samples was also employed to verify these results. According to gene relative expression results achieved by qRT-PCR and OS of LUAD patients, we analyzed expression correlations between genes and prognostic value of the core regulatory ceRNA axis. As Figure 9A presented, there existed a clear regulatory correlation between genes involved in the ceRNA axis. There were positive correlations between LINC00324 and GAB3/IKZF1 (*r* = 0.6289, *p* = 0.003/*r* = 0.5559, *p* = 0.0109). As for LINC00324 and miR-9-5p/miR-33b-5p, they possessed negative expression correlations (*r* = −0.4583, *p* = 0.0421/*r* = −0.5333, *p* = 0.0154). Similarly, miR-9-5p and GAB3 (*r* = −0.559, *p* = 0.0104) or miR-33b-5p and IKZF1 (*r* = −0.4981, *p* = 0.0254) also possessed negative expressions which suggested their potential regulatory relationships. Moreover, from survival analysis in Figures 9B–F, results suggested that high expression of LINC00324, GAB3, and IKZF1 predicted a good prognosis (*p* = 0.0147, 0.0048, and 0.0117, respectively) while miR-9-5p and miR-33b-5p appeared to predict a poor prognosis (*p* = 0.0739 and 0.0048, respectively), which were consistent with results analyzed from public datasets above.

Taken together, these findings demonstrated that the prognostic value and expression significance of key genes in core regulatory axis could be validated in external datasets.

## Discussion

Lung cancer is the most common type of cancer and remains the predominant cause of cancer deaths worldwide. LUAD is the most common histological subtype of lung cancer, with an average 5-year survival rate of 15% (Allemani et al., 2015). LUAD usually exhibits more morphological heterogeneity and relatively poor prognosis warranting the need for better treatment strategies (Zhang et al., 2018). In recent years, as research progresses, emerging evidences show that immunotherapy is becoming a promising treatment against LUAD. Although tumor immunotherapy, especially immune checkpoint blockade, has gradually revolutionized cancer treatment, there still remains a large portion of patients failed to benefit from the treatment (Horvath et al., 2020; Huang M.Y. et al., 2020). One of the key reasons for that was the obstruction of the TME which consisted of complicated cellular and molecular components (Chae et al., 2018; Seidel et al., 2018; Yang et al., 2019b; Lei et al., 2020). Recent evidences reveal that TAMs are the most abundant infiltrating immunosuppressive cells in the TME, playing a key role influencing efficacy of anti-tumor immunotherapy (Pérez-Ruiz et al., 2020). Therefore, understanding specific molecular mechanisms by which TAMs affect tumor immunotherapy is of great value for developing ideal treatment strategies for LUAD patients.

However, TAMs are highly plastic and heterogeneous in solid tumor (Ngiow and Young, 2020). Generally, Th1 cytokines such as lipopolysaccharide (LPS), interferon-γ (IFN-γ), and tumor necrosis factor-α induce macrophages into a M1-like phenotype, playing a role in antitumor inflammation. On the contrary, TAMs (M2-like) polarized by IL-4 and IL-13 play the opposite immunosuppression and pro-tumor function in the TME (Bohn et al., 2018). To our knowledge, there exists massive biomarkers of TAMs which represent function or phenotype of macrophage infiltrated in the tumors. Meanwhile, TAM-associated molecular markers appear to be showed a controversial prognostic value in pan-cancer patients for the expression of some markers are not absolutely specific (Zhao et al., 2019). Therefore, considering the heterogeneous phenotypes of TAMs, there are certain limitations making a distinction between M1-like and M2-like macrophages or predicting prognosis of patients by single molecular marker.

To this end, we aimed to comprehensively investigate broadly reported TAM signature genes to construct a precise prognostic risk model and further explore the underlying mechanism by which TAMs influence immunotherapy and tumor progression.

TAM-associated genes enrolled in this study were broadly reported as follows: The pan-macrophage marker CD68 is now generally utilized to identify TAMs in pathological specimen and has been reported associated with controversial prognostic value in patients with cancers including breast and ovarian cancer (Wang et al., 2018); CD163 as well as CD206 tend to be associated with worse clinical outcome and have been defined as M2-related markers combined with myeloid marker CD11b in most researches (Lu-Emerson et al., 2013; Xu et al., 2020); Cytokines and chemokines, including IL-10, TGFB1, CXCL8, and CCL17, play the immunosuppressive roles in the TME via recruiting regulatory T cells and myeloid-derived suppressor cells, serving as functional biomarker of TAMs (Cassetta and Pollard, 2018; Yang et al., 2019a); Metabolic enzymes, such as ARG1, IDO1, and ENTPD1, play key roles in regulating immune balance via various metabolic signaling pathways (Takenaka et al., 2019; Vitale et al., 2019); MMP14, a matrix metalloproteinase, has also been reported to induce TAM immunosuppression and could predict the prognosis of cancers (Alonso-Herranz et al., 2020); CD274, also known as PD-L1, contributing to the well-known PD-1/PD-L1 immune checkpoint theory, was involved in TAM immunosuppression (Noguchi et al., 2017).

The LASSO regression model is a frequently used statistical method for multicollinearity problems and has been demonstrated to be suitable for high dimensional data regression analysis. In this study, according to 13 TAM-related biomarkers described above, we constructed a TAM prognostic risk model containing 8 genes with the most prognostic valuable by LASSO cox regression in LUAD patients. Therefore, every patient would be assigned a risk score based on expression of 8 TAM-related genes according to the formula obtained from the risk model. As validated in TCGA- and GEO-validation cohort, the TAM prognostic risk model revealed an ideal prognostic value and it would be possible for us to distinguish between high and low prognostic risk for patients based on risk scores.

Afterward, in order to get a deeper insight into the specific molecular mechanisms inducing different survival prognosis between high and low-risk samples, comprehensive analysis about differentially expressed genes between these two groups was crucially needed. As a result, there revealed 381 DElncRNAs, 29 DEmiRNAs, and 1976 DEmRNAs between high- and low-risk groups. GO and KEGG pathway enrichment analysis were generally used to annotate gene sets and provide hints about functions and pathways participated by concerned genes.

Enrichment results showed that upregulated DEmRNAs in high-risk group were primarily involved in GO biological processes, such as “cell division,” “cell proliferation,” “DNA replication,” “DNA repair,” and KEGG pathways, such as “cell cycle,” “DNA replication” and “metabolic pathways.” It suggests that there exist more genes related to tumor development and progression in TAMs high-risk patients. In the meantime, it was noteworthy that downregulated genes in high-risk group were mainly enriched in “immune response,” “phagocytosis,” and “cytokine-cytokine receptor interaction.” These results suggest that differentially expressed genes between high-risk and low-risk groups played an imperative role in tumor development and immunosuppression which is exactly consistent with the immunosuppression of TAMs in the TME and this also illustrates the validity of our risk model.

Next, in order to further narrow the focus on specific genes, we employed WGCNA, ceRNA and PPI network analysis with these dysregulated genes. WGCNA is commonly used to enrich genes with similar expression patterns into modules associated with clinical characters. In this study, we identified two interested modules containing a large number of DEGs associated with risk score and TNM stage. Of note, GO and KEGG analysis showed that turquoise module had a significant correlation with immunosuppression while blue module tended to be associated with tumor development, which was consistent with their upregulated or downregulated patterns. As reported before, ceRNAs played an essential role in regulating interactions among different varieties of RNAs and were involved in progression and immune infiltration in multiple kinds of tumors (Zhang K. et al., 2020). We constructed ceRNA networks in concerned WGCNA modules through predictive algorithm. Prediction of complexes in PPI networks is significant for understanding the principles of cellular organization and function. In this study, we performed PPI analysis in order to explore intrinsic relationships between mRNAs and identify hub genes which may play important role in prognosis in ceRNA networks. By synthesizing information of PPI analysis and survival analysis, we have eventually identified a core regulatory axis: LINC00324/miR-9-5p (miR-33b-5p)/GAB3 (IKZF1) which may play a pivotal role in regulating TAM risk and the prognosis in LUAD patients.

In reviewing the literature, genes in the core regulatory axis function through different approaches influencing tumor progression and the immune microenvironment.

Gab3 is a kind of adaptor proteins expressed mainly in hematopoietic cells, such as lymphocytes and bone marrow-derived macrophages, functioning as scaffolding and docking molecules. The role of Gab3 in immune cells is incompletely understood. Relationship between Gab3 and macrophages was firstly reported in 2002. Rohrschneider et al., reported that Gab3 was tyrosine phosphorylated after macrophage colony stimulating factor receptor stimulation and then accelerated macrophage morphological differentiation (Liu and Rohrschneider, 2002; Wolf et al., 2002). However, further analysis demonstrated that hematopoiesis in mice lacking Gab3 was not impaired and macrophages developed in normal numbers exhibited normal function (Seiffert et al., 2003). Colucci recently indicated that Gab3 may promote expansion and function of NK cells through MAPK-ERK pathway (Colucci, 2019). Sliz et al. (2019) also found that knockout of Gab3 induced defective uNK cell expansion, suggesting that Gab3 was a key component required for cytokine-mediated NK cells priming and expansion that is essential for antitumor responses. Gab3 plays a controversial role in immune system. Cheng et al. (2018) indicated that Gab3 expression was upregulated by IFN and Gab3 demonstrated antiviral effects through enhancing IFN response and innate immune activation. However, Wang et al. (2019) illustrated the importance of Gab2/3 in controlling macrophages and CD8^+^ T cells activation and suppressing chronic colitis. Besides, several researches recently suggested that Gabs acted as tumor-promoting molecule in colorectal, glioma, and ovarian cancer (Jia et al., 2017; Xiang et al., 2017; Berkel and Cacan, 2020). IKZF1, same as Gab3, plays a controversial role in immune system. Ikaros is a member of the kruppel family of zinc finger DNA-binding proteins encoded by IKZF1, functioning as a master regulator of hematopoiesis and the immune system. As reported, Ikaros was widely expressed in tumors but performed anti-tumor or pro-tumor function in different researches (Dhanyamraju et al., 2020). As for immune cells, Dumortier et al. (2003) reported that Ikaros positively regulates early neutrophil differentiation. While, Singhal et al. (2016) demonstrated that Ikaros affected anti-tumor response through inhibiting APC-like neutrophils. As for macrophage, Cho et al. (2008) demonstrated that Ikaros acted as a negative regulator on LPS/IFN-γ-induced iNOS expression in macrophages. Moreover, Oh et al. (2018) described unexpected dual repressor and activator functions for Ikaros in the LPS response of murine macrophages. Of note, Chen et al. (2018) reported that IKZF1 overexpression promoted immune infiltration in several tumor types, and enhanced the efficacy of anti-PD1 and anti-CTLA4 treatment. Besides, non-coding RNAs in our study, including LINC00324 (Ni et al., 2019; Zhang M. et al., 2020), miR-9-5p (Ma et al., 2020; Wang et al., 2020), and miR-33b-5p (Huang G. et al., 2020; Ni et al., 2020), were also reported in literatures to be associated with the prognosis of various cancer types.

In our study, upregulation of LINC00324, GAB3 as well as IKZF1 in TAM low-risk group could predict a better prognosis, suggesting the potential anti-tumor immunology role in the TME. However, miR-9-5p and miR-33b-5p present as pro-tumor molecules whose immunosuppression may be achieved through regulating expression of mRNAs they targeted. To our knowledge, although there have been lots of valuable researches about these genes, how do they influence the prognosis of LUAD patients through TAMs has not been reported to date. In our study, core regulatory axis obtained from TAM risk model showed an ideal prognostic value, suggesting that these genes could influence the prognosis of LUAD through regulating polarization or infiltration of TAMs.

Several limitations need to be acknowledged regarding the present study. Firstly, findings and results in this study were indirect because we explored how TAMs potentially influenced the prognosis of LUAD patients mainly through utilizing bioinformatic approaches analyzing public datasets with TAMs biomarkers. In addition, in the validation part, the small size of clinical samples limited our validation power of the prognostic value and correlation between genes involved in the core regulatory axis. Therefore, these preliminary findings and specific deep mechanism of this axis deserve further direct experimental studies.

## Conclusion

In conclusion, we utilized bioinformatic approaches analyzing public datasets to explore how TAMs potentially influence the prognosis of LUAD patients. Eventually, we have identified a core regulatory axis: LINC00324/miR-9-5p (miR-33b-5p)/GAB3 (IKZF1) which may play a pivotal role in regulating TAM risk and prognosis in LUAD patients. Although the current study is mainly based on public data analysis through bioinformatic approaches, findings in this work contribute to a better understanding of TAM-associated immunosuppression in the TME and provide novel targets and regulatory pathways for anti-tumor immunotherapy. In the future, we will employ more convincing experimental researches to confirm this core regulatory ceRNA axis in further studies.

## Data Availability Statement

Publicly available datasets were analyzed in this study. This data can be found here: https://tcga-data.nci.nih.gov/tcga/, https://www.ncbi.nlm.nih.gov/geo/, accession: GSE72094.

## Author Contributions

LZ, KZ, SL, LY, YZ, and JW designed, edited, and led out this study. LZ, KZ, and SL collected materials and conducted data analysis. RZ, YY, QW, and SZ raised critical discussions of the results and provided technical support. All authors contributed to the writing and editing of the manuscript and approved the final draft of the manuscript.

## Conflict of Interest

The authors declare that the research was conducted in the absence of any commercial or financial relationships that could be construed as a potential conflict of interest.

## Footnotes

1 https://tcga-data.nci.nih.gov/tcga/

2 https://www.ncbi.nlm.nih.gov/geo/

3 http://www.geneontology.org

4 http://www.kegg.jp/

5 http://www.microrna.org/

6 http://www.targetscan.org/

7 http://129.206.7.150/

8 https://genie.weizmann.ac.il/pubs/mir07/mir07_exe.html

9 http://string-db.org/
