# Supplementary material for: Identification of a ceRNA Network in Lung Adenocarcinoma Based on Integration Analysis of Tumor-Associated Macrophage Signature Genes
Source: Front Cell Dev Biol. 2021 Mar 2;9:629941. doi: 10.3389/fcell.2021.629941 (PMC7960670; doi:10.3389/fcell.2021.629941)
Supplement: Supplementary file 2 [file Table_1.docx]

**Supplementary Table S1. The detail information for 13 TAMs related genes.**

| **Gene ID** | **Gene name** | **Classification** | **Description** |
| --- | --- | --- | --- |
| **CD68** | CD68 | adhesive molecule | CD68 is a commonly used marker for macrophage regardless of their polarization(Wang et al., 2018) |
| **MRC1**  **(CD206)** | mannose receptor C-type 1 | adhesive molecule | CD206 is mainly used for the detection of polarized M2 TAMs(Lu-Emerson et al., 2013) |
| **CD163** | CD163 | scavenger receptor | CD163 is mainly used for the detection of polarized M2 TAMs(Lu-Emerson et al., 2013) |
| **ITGAM**  **(CD11b)** | integrin subunit alpha M | complement receptor | CD11b is mainly used for identification myeloid cells and regulates macrophage polarization(Lu-Emerson et al., 2013) |
| **IL10** | interleukin 10 | anti-inflammatory cytokine | play various immunosuppressive roles in TME(Cassetta and Pollard, 2018) |
| **TGFB1** | transforming growth factor beta 1 | anti-inflammatory cytokine | play various immunosuppressive roles in TME(Cassetta and Pollard, 2018) |
| **CCL17** | C-C motif chemokine ligand 17 | chemokine | immunosuppressive chemokine, recruitment for Tregs(Cassetta and Pollard, 2018) |
| **CXCL8**  **(IL8)** | C-X-C motif chemokine ligand 8 | chemokine | immunosuppressive chemokine, angiogenesis, survival signaling for cancer stem cells, recruitment for MDSCs(Cassetta and Pollard, 2018) |
| **ARG1** | arginase 1 | metabolic enzyme | arginase catalyzes the hydrolysis of arginine to ornithine and urea(Vitale et al., 2019) |
| **IDO1** | indoleamine 2,3-dioxygenase 1 | metabolic enzyme | a heme enzyme that catalyzes the first and rate-limiting step in tryptophan catabolism to N-formyl-kynurenine(Vitale et al., 2019) |
| **ENTPD1**  **(CD39)** | ectonucleoside triphosphate diphosphohydrolase 1 | metabolic enzyme | ecto-enzymes that favor extracellular adenosine accumulation(Takenaka et al., 2019) |
| **MMP14** | matrix metallopeptidase 14 | matrix metallopeptidase | promote tumor cell migration and invasion(Alonso-Herranz et al., 2020) |
| **CD274**  **(PD-L1)** | programmed death ligand 1 | immunosuppressive membrane protein | ligand of PD1, immune checkpoint molecule(Noguchi et al., 2017) |

Alonso-Herranz, L., Sahún-Español, Á., Paredes, A., Gonzalo, P., Gkontra, P., Núñez, V., et al. (2020). Macrophages promote endothelial-to-mesenchymal transition via MT1-MMP/TGFβ1 after myocardial infarction. *Elife* 9. doi: 10.7554/eLife.57920.

Cassetta, L., and Pollard, J.W. (2018). Targeting macrophages: therapeutic approaches in cancer. *Nat Rev Drug Discov* 17(12)**,** 887-904. doi: 10.1038/nrd.2018.169.

Lu-Emerson, C., Snuderl, M., Kirkpatrick, N.D., Goveia, J., Davidson, C., Huang, Y., et al. (2013). Increase in tumor-associated macrophages after antiangiogenic therapy is associated with poor survival among patients with recurrent glioblastoma. *Neuro Oncol* 15(8)**,** 1079-1087. doi: 10.1093/neuonc/not082.

Noguchi, T., Ward, J.P., Gubin, M.M., Arthur, C.D., Lee, S.H., Hundal, J., et al. (2017). Temporally Distinct PD-L1 Expression by Tumor and Host Cells Contributes to Immune Escape. *Cancer Immunol Res* 5(2)**,** 106-117. doi: 10.1158/2326-6066.Cir-16-0391.

Takenaka, M.C., Gabriely, G., Rothhammer, V., Mascanfroni, I.D., Wheeler, M.A., Chao, C.C., et al. (2019). Control of tumor-associated macrophages and T cells in glioblastoma via AHR and CD39. *Nat Neurosci* 22(5)**,** 729-740. doi: 10.1038/s41593-019-0370-y.

Vitale, I., Manic, G., Coussens, L.M., Kroemer, G., and Galluzzi, L. (2019). Macrophages and Metabolism in the Tumor Microenvironment. *Cell Metab* 30(1)**,** 36-50. doi: 10.1016/j.cmet.2019.06.001.

Wang, L., Zhang, C., Zhang, Z., Han, B., Shen, Z., Li, L., et al. (2018). Specific clinical and immune features of CD68 in glioma via 1,024 samples. *Cancer Manag Res* 10**,** 6409-6419. doi: 10.2147/cmar.S183293.
