# Supplementary material for: Identification of a ceRNA Network in Lung Adenocarcinoma Based on Integration Analysis of Tumor-Associated Macrophage Signature Genes
Source: Front Cell Dev Biol. 2021 Mar 2;9:629941. doi: 10.3389/fcell.2021.629941 (PMC7960670; doi:10.3389/fcell.2021.629941)
Supplement: Supplementary file 4 [file Table_3.DOCX]

| **Module** | **Gene number** | **Relations to clinical characters** | | | | | | | | | |
| --- | --- | --- | --- | --- | --- | --- | --- | --- | --- | --- | --- |
|  |  | **high-RS** | **low-RS** | **Stage** | **T** | **N** | **M** | **age** | **gender** | **race** | **RFS** |
| **turquoise** | **341** | R = -0.48  *p* = 3e-13 | R = 0.48  *p* = 3e-13 | R = -0.2  *p* = 0.004 | R = -0.24  *p* = 7e-04 | R = -0.1  *p* = 0.1 | R = -0.21  *p* = 0.003 | R = 0.21  *p* = 0.002 | R = 0.22  *p* = 0.001 | R = 0.078  *p* = 0.3 | R = 0.0037  *p* = 1 |
| **blue** | **264** | R = 0.47  *p* = 1e-12 | R = -0.47  *p* = 1e-12 | R = 0.29  *p* = 2e-05 | R = 0.12  *p* = 0.09 | R = 0.2  *p* = 0.004 | R = 0.089  *p* = 0.2 | R = -0.2  *p* = 0.005 | R = -0.17  *p* = 0.01 | R = -0.068  *p* = 0.3 | R = 0.011  *p* = 0.9 |
| **brown** | **161** | R = -0.3  *p* = 1e-05 | R = 0.3  *p* = 1e-05 | R = -0.22  *p* = 0.002 | R = -0.2  *p* = 0.005 | R = -0.19  *p* = 0.007 | R = -0.11  *p* = 0.1 | R = 0.088  *p* = 0.2 | R = 0.15  *p* = 0.03 | R = 0.078  *p* = 0.3 | R = -0.08  *p* = 0.3 |
| **yellow** | **126** | R = -0.44  *p* = 3e-11 | R = -0.44  *p* = 3e-11 | R = -0.17  *p* = 0.02 | R = -0.19  *p* = 0.008 | R = -0.15  *p* = 0.03 | R = -0.26  *p* = 2e-04 | R = 0.15  *p* = 0.03 | R = 0.19  *p* = 0.006 | R = -0.0055  *p* = 0.9 | R = -0.094  *p* = 0.2 |

**Supplementary Table S3. Detail information about concerned WGCNA modules**
