# Supplementary material for: Identification of a ceRNA Network in Lung Adenocarcinoma Based on Integration Analysis of Tumor-Associated Macrophage Signature Genes
Source: Front Cell Dev Biol. 2021 Mar 2;9:629941. doi: 10.3389/fcell.2021.629941 (PMC7960670; doi:10.3389/fcell.2021.629941)
Supplement: Supplementary file 6 [file Table_5.docx]

**Supplementary Table S5.**

**Primers used in this study.**

| **Gene name** | **Primer sequences** |
| --- | --- |
| miR-9-5p-F | CGCGTCTTTGGTTATCTAGCTGTATGA |
| miR-33b-5p-F | GGTGCATTGCTGTTGCATTGC |
| LINC00324 | F: TGTGGATGACAGTGTTCGGG  R: ACGCTGACCAGAAACCGTAG |
| GAB3 | F: GCAACCCCGATGTCTTGGAG  R: ACTCGCTGAGGTCTATCACCC |
| IKZF1 | F: CATCAGCCCGATGTACCAGC  R: CCTCGTTGTTGCTCTCGGT |
| GAPDH | F: ATGGAGAAGGCTGGGGCTC  R: AAGTTGTCATGGATGACCTTG |

Note: Universal U6 Primer F and Universal PCR Primer R were provided by miRNA First Strand cDNA Synthesis Tailing Reaction Kit (B532451, Sangon Biotech, Shanghai, China) and the sequence information is commercially protected.
