# Supplementary material for: Identification of a ceRNA Network in Lung Adenocarcinoma Based on Integration Analysis of Tumor-Associated Macrophage Signature Genes
Source: Front Cell Dev Biol. 2021 Mar 2;9:629941. doi: 10.3389/fcell.2021.629941 (PMC7960670; doi:10.3389/fcell.2021.629941)
Supplement: Supplementary file 7 [file Table_6.docx]

**Supplementary Table S6.**

Univariate and multivariate analyses of confounders and TAMs risk score for prognosis of LUAD patients.

| Variables | Univariate cox |  |  | Multivariate cox |  |  |
| --- | --- | --- | --- | --- | --- | --- |
|  | HR | CI 95 | *p* value | HR | CI 95 | *p* value |
| Age | 0.81 | 0.46-1.42 | 0.455 | 0.68 | 0.34-1.36 | 0.275 |
| Gender | 0.74 | 0.46-1.19 | 0.212 | 1.04 | 0.6-1.81 | 0.883 |
| Race | 0.77 | 0.35-1.72 | 0.529 | 0.73 | 0.27-1.96 | 0.534 |
| Stage | 1.98 | 1.2-3.29 | 0.008 | 1.63 | 0.9-2.97 | 0.109 |
| Smoking | 0.83 | 0.44-1.57 | 0.571 | 0.74 | 0.35-1.56 | 0.427 |
| TAMs risk score | 2.2 | 1.36-3.56 | 0.001 | 1.9 | 1.08-3.33 | 0.025 |
