# Supplementary material for: Identification of a ceRNA Network in Lung Adenocarcinoma Based on Integration Analysis of Tumor-Associated Macrophage Signature Genes
Source: Front Cell Dev Biol. 2021 Mar 2;9:629941. doi: 10.3389/fcell.2021.629941 (PMC7960670; doi:10.3389/fcell.2021.629941)

Supplementary Figure S1. Identification of outlier samples by cluster analysis based on DElncRNAs and DEmRNAs.

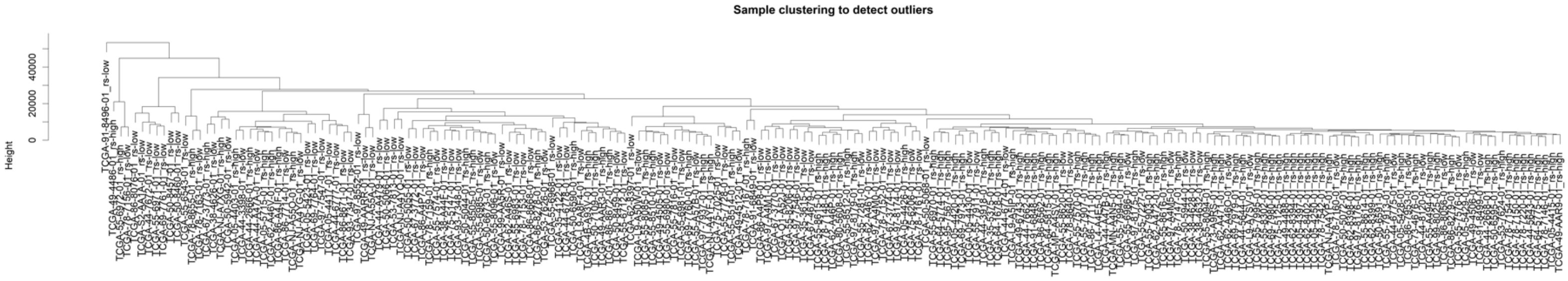

Supplement: Supplementary file 1 [file Data_Sheet_1.PDF]
